# Supplementary material for: Prognostic significance of total choline on in-vivo proton MR spectroscopy for prediction of late recurrence in patients with hormone receptor-positive, HER2-negative early breast cancer
Source: PLoS One. 2025 Jan 2;20(1):e0311012. doi: 10.1371/journal.pone.0311012 (PMC11695009; doi:10.1371/journal.pone.0311012)
Supplement: Attachment — Submitted filename: Response to Reviewer.docx [file pone.0311012.s001.docx]

Dear Editor,

Reviewer #1

***Comment 1:***

-line 134: authors should make clear that the Ki67% threshold of 20% is defining luminal A from luminal B tumors.

**Reply 1:** We clarify it on manuscript in Study variables and pathologic analysis park, line 141-143

The cutoff for the Ki-67 index was set at 20%, which can distinguish between Luminal A and Luminal B breast cancer subtypes.

***Comment 2:***

-line 178: if this was a retrospective study,it means that tCho was part of the routine of breast MRI imaging which is not the case in most institutions. Are all patients receiving pre operative breast MRI in this center, also receive MRS? can we have more explanation concerning this institutional policy?

**Reply 2:** We clarify it on manuscript in Study population part, line 184-186

This was a single-center retrospective cohort study, in which all breast cancer patients from March 2011 to July 2014 underwent initial MRI along with in vivo proton MRS for research and diagnostic purposes.

Reviewer #2

***Comment 1:***

1. Introduction

Add a brief mention of CHPT1 and its role in choline metabolism to set up the biological context for the results.

**Reply 1:** I have inserted the relevant content into the Introduction section. Line 73-78

A recent study of the relationship between ERα and choline metabolism found that ERα directly regulated the gene encoding Cho phosphotransferase 1 (CHPT1), an enzyme necessary for estrogen to affect Cho metabolism, including increased phosphatidylcholine synthesis [17]. This finding suggests that choline metabolism is involved in the development and/or progression of hormone receptor (HR)-positive, relatively non-aggressive breast cancer, as opposed to the more aggressive subtypes of breast cancer studied previously.

***Comment 2:***

2. Results

Table 3 shows the relationship between total choline (tCho) and prognostic factors like histologic and nuclear grades. Besides highlighting statistically significant results (p < 0.05), it is valuable to discuss findings with clinically meaningful trends, even if they lack statistical significance. Including these insights can enhance the analysis and improve readers' understanding of the data.

**Reply 2:** The mentioned content was written in the Results and Discussion sections of the previous draft. Line 210-213(result), Line 262-268(Discussion)

***Comment 3:***

3. Discussion

The manuscript highlights a stronger association between tCho and late recurrence than early recurrence. An analysis of the potential reasons for this difference would enhance the interpretation of the findings.

**Reply 3:** I have added and revised a paragraph discussing the association between CHPT1, tamoxifen-resistant cells, and late recurrence.

Previous studies have also reported an association between CHPT1 and ERα. For example, the CHPT1 gene, which is directly regulated by ERα, was required for estrogen-induced effects on Cho metabolism, including increased phosphatidylcholine (PtdCho) synthesis [17]. Additionally, immunohistochemical (IHC) analysis showed that the expression of CHPT1 is higher in breast cancer cells than in normal breast tissue and is higher in ER-positive than in ER-negative breast cancer [17]. The present study showed that survival rates following late recurrence were associated with tCho levels. CHPT1 also plays a role in the invasion of tamoxifen-resistant breast cancer cells, with tamoxifen-resistant LCC2 cells exhibiting greater invasiveness than tamoxifen-sensitive MCF7 cells under CHPT1 expression [17]. Moreover, CHPT1 depletion resulted in stronger suppression of invasion and metastasis by LCC2 cells than by MCF7 cells [17]. ER-positive breast cancer has often been associated with late recurrence [21], with extended hormone therapy administered to many patients with ER-positive breast cancer to reduce the risk of recurrence [22]. Adherence to hormone therapy, however, often decreases after 5 years [23], with many patients not receiving extended hormone therapy. This environment of minimal residual disease (MRD) may function similarly to tamoxifen-resistant breast cancer cells, potentially increasing CHPT1 activity.

Thank you.

Sincerely,

Hyun Jik Kim
